# The impact of airway assistants on prehospital endotracheal intubations – a subgroup analysis of data from anaesthesiologist-staffed helicopter critical care teams

**DOI:** 10.1186/s13049-025-01515-y

**Published:** 2025-11-25

**Authors:** Jacob Broms, Mattias Günther, Christer Svensén, Andreas Krüger, Leif Rognås, Mikael Gellerfors

**Affiliations:** 1 Department of Clinical Science and Education, Södersjukhuset, Karolinska Institutet, Stockholm, Sweden; 2 Department of Emergency Medicine and Prehospital Services, St. Olav’s University Hospital, Trondheim, Norway; 3 Department of Research and Development, Norwegian Air Ambulance Foundation, Oslo, Norway; 4 Danish Air Ambulance, Skive, Denmark; 5 Department of Physiology and Pharmacology, Section for Anaesthesiology and Intensive Care Medicine, Karolinska Institutet, Stockholm, Sweden; 6 Department of Perioperative Medicine and Intensive Care, Karolinska University Hospital, Stockholm, Sweden; 7 Swedish Air Ambulance, Mora, Sweden; 8 Ambulance Helicopter and Rapid Response Car, AISAB, Stockholm, Sweden

**Keywords:** Airway management, Emergency medical services, Intubation, Intratracheal, Airway assistant

## Abstract

**Background:**

Advanced airway management in the prehospital environment is a technically demanding and high-stakes procedure requiring effective team collaboration. While operator skill is often emphasized, few studies have examined whether an airway assistant’s professional background influences intubation outcomes. This subgroup analysis of prehospital advanced airway management data aimed to evaluate whether the airway assistant profession affects first-pass success and complication rates during prehospital drug-assisted endotracheal intubation performed by anaesthesiologist-staffed Scandinavian helicopter emergency medical services.

**Methods:**

This retrospective subgroup analysis included 422 patients from 12 helicopter emergency medical services across Denmark, Norway, and Sweden between March 2020 and September 2021. The primary outcome was the first-pass success rate, which was defined as successful endotracheal intubation on the first attempt. The main exposure variable was the airway assistant’s professional category, *anaesthetist* (anaesthesiologist or nurse anaesthetist) or *non-anaesthetist* (paramedic, other physician, other nurse, or other healthcare provider). The secondary outcomes included intubation-related complications. Binary logistic regression was used to assess associations between the assistant profession and first-pass success or complication rates, adjusting for predefined covariates.

**Results:**

Among the 422 drug-assisted intubations, 143 (33.9%) involved anaesthetist assistants and 279 (66.1%) involved non-anaesthetists. First-pass success was similar between groups (88.1% vs. 87.8%, p = 1.000), with an adjusted odds ratio of 1.05 (95% CI 0.54–2.12). The overall complication rate was 10.7%, with no significant difference between the groups (8.4% vs. 11.8%, p = 0.32; adjusted odds ratio 1.79, 95% CI 0.66–5.39). Hypoxia was more common in the anaesthetist-assisted group (7.7% vs. 3.9%, p = 0.00115), but this may reflect case-mix differences. No other significant differences were found in procedural performance or outcomes.

**Conclusions:**

In this large, multicentre observational study, the airway assistant profession was not independently associated with first-pass success or complication rates. These findings suggest that in mature helicopter emergency medical services with experienced anaesthesiologists and standardized protocols, team functioning and structured processes may outweigh individual assistant backgrounds in determining airway management success.

**Trial registration (clinical trial number):**

NCT04206566 (first record registered 2019–12-18).

## Background

Advanced airway management in the prehospital environment is a technically demanding and high-stakes procedure. While drug-assisted tracheal intubation is frequently performed by anaesthesiologist-staffed helicopter emergency medical services (HEMS) in Scandinavia, successful performance requires effective collaboration between multiple team members. Crew resource management (CRM), human factors, and team composition, including the assistant’s competence and experience, may therefore influence procedural quality and outcomes [1, 2].

Compared with the controlled setting of the operating theatre or emergency department in-hospital, the prehospital environment introduces unique challenges to airway management. These include unpredictable and often adverse environmental conditions, a lack of backup resources, and the need for tasks such as extrication, resuscitation, and scene safekeeping to be performed in parallel. Under such conditions, prehospital tracheal intubation becomes not only a technical task but also a team-based intervention that depends on pre-established roles, good communication, and task prioritization. The ability to deliver safe and effective drug-assisted intubation under these constraints reflects not only on individual skills but also the functioning of the team as a whole.

In-hospital guidelines, such as those from the Difficult Airway Society (DAS) and the Project for Universal Management of Airways (PUMA), emphasize structured team roles, shared mental models, and the critical contribution of the assistant to airway safety [2, 3]. Leading prehospital systems such as the Greater Sydney HEMS and London HEMS have incorporated similar principles into standard operating procedures (SOP), using checklists, procedural bundles, and pre-allocated team roles to improve performance in emergency anaesthesia [3, 4]. In Scandinavia, the SSAI clinical practice guideline for prehospital airway management also endorses structured preparation, cognitive aids, and assistant-task allocation [5].

Despite the known importance of teamwork in airway management, most clinical studies in this area focus solely on the primary operator, often categorizing outcomes by whether the tracheal intubator is a physician, paramedic, or nurse. Fewer studies have considered the role of the airway assistant, operator two, even though assistants often play a crucial role in delivering and managing drugs, preparing and handing over the airway equipment, monitoring vital signs, and helping confirm correct tube placement. This oversight is particularly relevant in HEMS systems where both the operator and the assistant are often highly skilled. In such contexts, the division of roles may be overlapping, and performance can depend more on mutual competence and shared expectations than on fixed hierarchy.

To date, no large-scale clinical study has evaluated whether an airway assistant’s profession or competence influences first-pass success (FPS) or complication rates in the prehospital setting. This gap is particularly evident in high-performing HEMS systems where both the operator and the assistant may be highly experienced. By leveraging prospective data collected across 12 Scandinavian anaesthesiologist-staffed HEMS bases, this study aims to address that gap by exploring whether the assistant category is associated with process and safety outcomes in prehospital tracheal intubations.

## Methods

This is a retrospective subgroup analysis of data from the PHASTER study (Prehospital Airway management – Success and complications using a Template for Enhanced Reporting), a prospective multicentre observational study conducted between March 2020 and September 2021. The study involved 12 anaesthesiologist-staffed HEMS units. Four from Denmark, who were staffed by a consultant-level anaesthesiologist, a paramedic and a pilot, four from Norway, who were staffed by a consultant-level anaesthesiologist, a paramedic or a nurse, and a pilot, and four from Sweden who were staffed by a consultant-level anaesthesiologist, a nurse anaesthetist, and one or two pilots. All the participating HEMS critical care teams adhered to their original structured local protocols for drug-assisted tracheal intubation and prehospital emergency anaesthesia since this was an observational study with no new intervention applied.

The PHASTER study was pre-registered at ClinicalTrials.gov (NCT04206566) in 2019–12–18 and had appropriate ethical approvals in Sweden (Dnr 2019–04943), Norway (REK 2019–63065), and Denmark (concluded to follow local regulations, hence no ethical permit was required). This subgroup analysis was approved by the Swedish Ethical Review Authority (Etikprövningsmyndigheten) (Dnr 2023–03224-01). This study was a retrospective observational analysis using de-identified data collected according to the internationally standardized Utstein-style template for prehospital advanced airway management [6]. In accordance with national regulations and ethical guidelines, informed consent was waived by the relevant regional ethics committee because the study posed no risk to subjects, did not impact patient care, and used previously recorded data in a fully anonymized format. The dataset and general methodology have previously been described in detail in Broms et al. [7]

All drug-assisted tracheal intubations attempted by the participating critical care teams were eligible for inclusion in the study. Drug-assisted intubation was defined as the administration of a sedative together with a neuromuscular blocking agent, with or without an analgesic, followed by attempted tracheal intubation. Intubations performed without the use of drugs (e.g. during cardiopulmonary resuscitation) were excluded.

Data were collected via a structured case report form (CRF) developed in accordance with the updated Utstein-style template for prehospital airway studies [6]. The CRF focused specifically on drug-assisted advanced airway management in the prehospital setting and captured variables related to team composition, procedural context, airway characteristics, operator experience, and clinical outcomes.

The primary endpoint of this subgroup analysis was FPS, defined as successful placement of the tracheal tube on the first laryngoscopic attempt. The main exposure variable of the cohorts compared was the airway assistant’s professional category, *anaesthetist* (anaesthesiologist or nurse anaesthetist) or *non-anaesthetist* (paramedic, other physician, other nurse, or other healthcare provider). The secondary endpoints included documented complications, the presence of difficult airway predictors, aggravated external conditions, and patient variables. The complications were classified via predefined categories aligned with international consensus definitions, including hypoxia, hypotension, bradycardia, cardiac arrest, oesophageal or bronchial intubation, aspiration, and dental trauma [6].

**Fig. 1:**
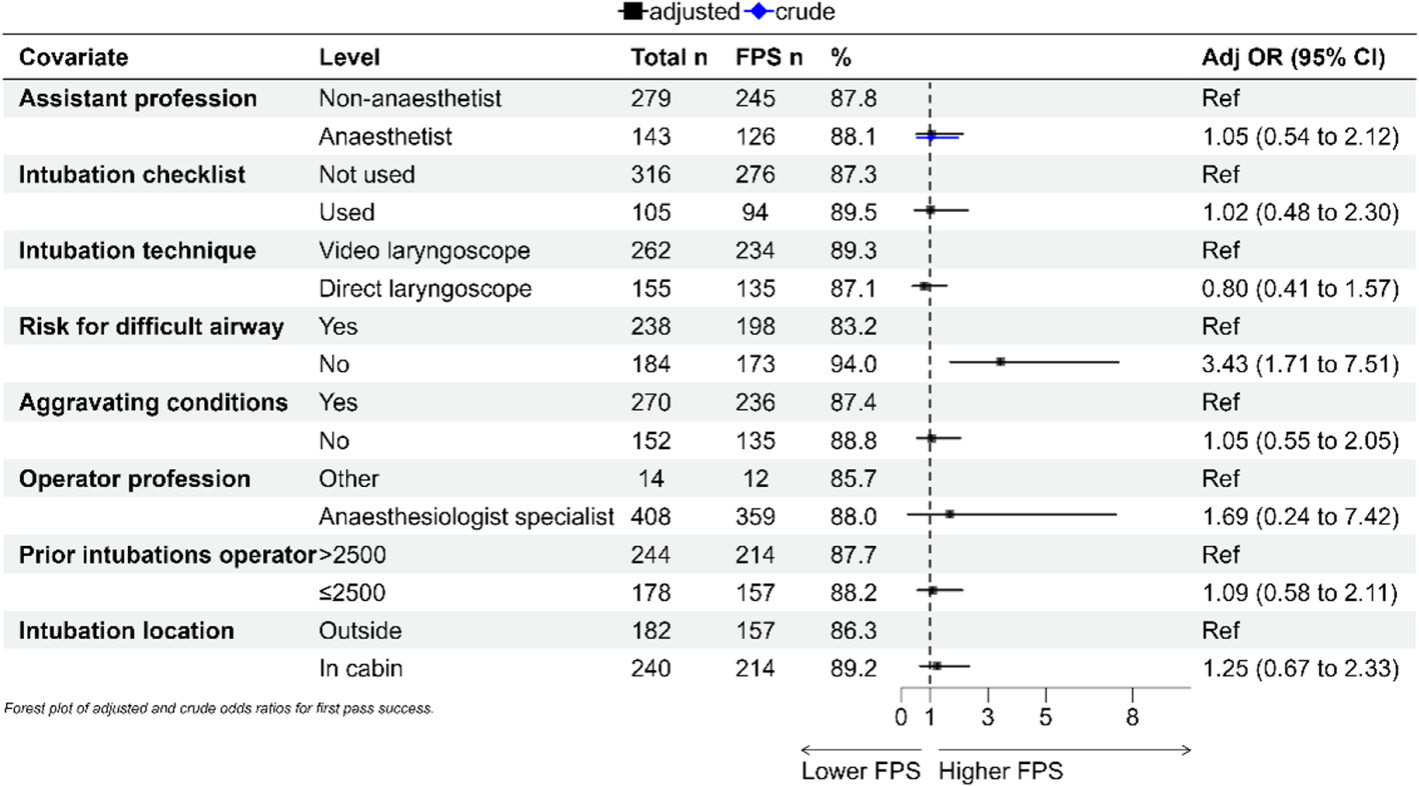
Associations between First Pass Success and Airway Assistant Profession. Adjusted odds ratio (Adj OR) are adjusted for all covariates. (FPS, first pass success rate)

**Fig. 2:**
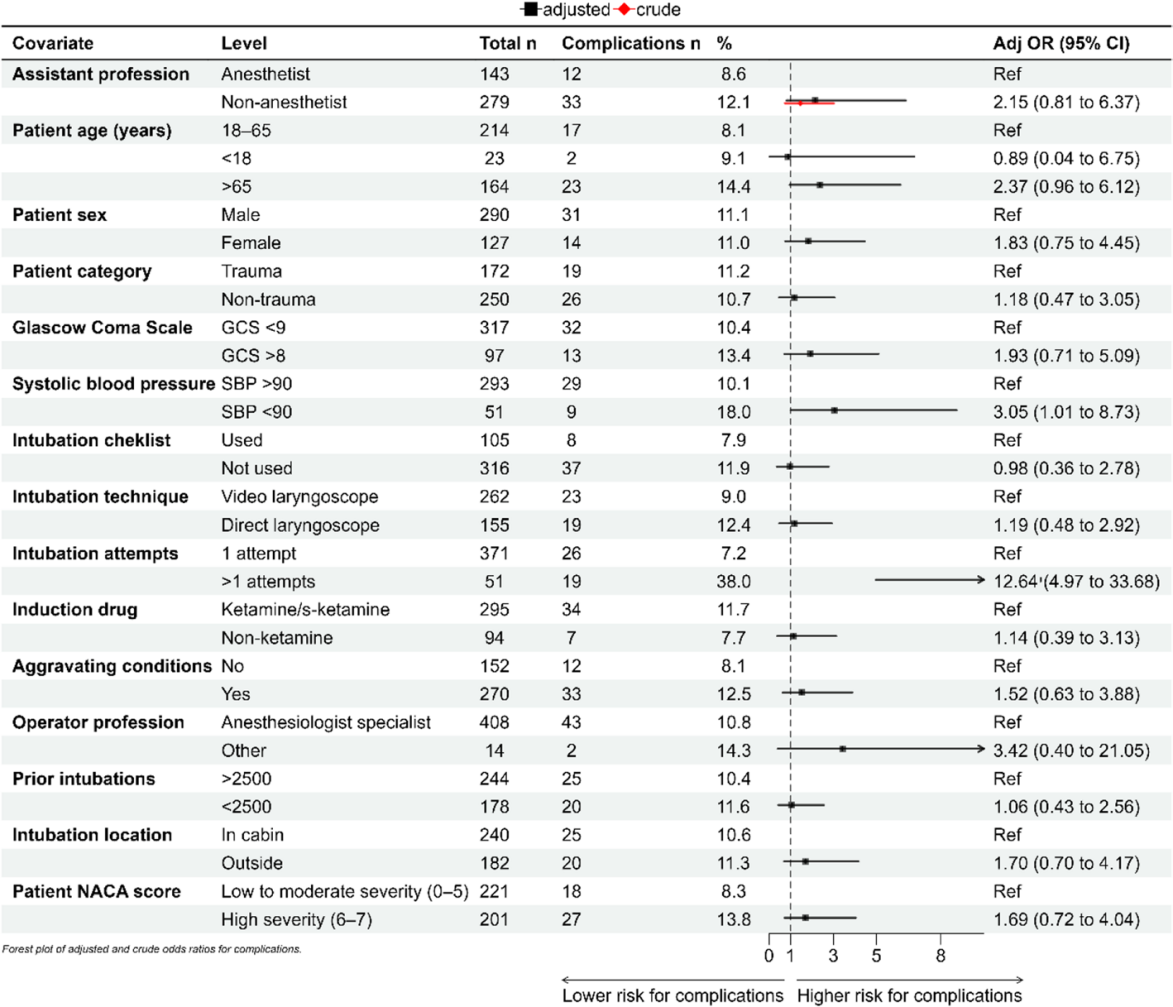
Associations between Complications and Airway Assistant Profession. Adjusted odds ratio (Adj OR) are adjusted for all covariates

The descriptive statistics in Tables 1, 2, and 3 are presented as the means and standard deviations (SD) for normally distributed continuous variables, and as medians with interquartile ranges (IQR) for non-normally distributed or ordinal data. Normality was checked via the Shapiro–Wilk’s test. Categorical variables are reported as absolute numbers and percentages. For group comparisons, chi-square tests were used for binary categorical variables, Fisher’s exact test was applied in cases of small, expected frequencies, and the Mann–Whitney U test was used for ordinal or non-normally distributed continuous variables. Independent t-tests were applied for continuous variables with approximately normal distributions. All tests were two-tailed, and p-values < 0.05 were considered statistically significant. Binary logistic regression was used to evaluate the associations between the assistant category and two separate outcomes: FPS and complications. Each outcome was analysed in a separate model, adjusting for relevant confounders (presented in Figs. 1 and 2). Covariates were selected a priori based on clinical relevance and supported by existing literature. Predictors such as operator experience, difficult airway, and aggravating conditions are established factors influencing FPS [8]. Checklist use has been shown to improve FPS and reduce hypoxia [9]. Intubation location and its possible correlation with FPS was described in the first article from the PHASTER-study [7]. The use of video laryngoscopy was included given its documented effect on FPS in critically ill patients [10, 11]. Adjusted odds ratios (ORs) with 95% confidence intervals (CIs) were reported, with crude ORs reported for airway assistant category. Model assumptions were assessed, and model fit was evaluated via appropriate diagnostics. Statistical analyses were performed via RStudio (version 2022.02.3). This study is reported in accordance with the Strengthening the Reporting of Observational Studies in Epidemiology (STROBE) statement [12].

**Table 1:** Baseline Characteristics by Assistant Type (Anaesthetist (anaesthesiologist or nurse anaesthetist) vs Non-anaesthetist (paramedic, emergency medicine physician, nurse, or other healthcare provider)

Airway assistant	Anaesthetist (*n* = 143)	Non-anaesthetist (*n* = 279)	*p*	Overall (*n* = 422)
**Operator (%)**				
Anaesthesiologist (specialist physician)	131 (91.6)	277 (99.3)	< 0.001	408 (96.7)
Others	12 (8.4)	2 (0.7)		14 (3.3)
**Experience of tracheal intubations (%)**				
≤ 250	1 (0.7)	0 (0.0)	0.227	1 (0.2)
251–1000	11 (7.7)	35 (12.5)		46 (10.9)
1001–2500	44 (30.8)	87 (31.2)		131 (31.0)
> 2500	87 (60.8)	157 (56.3)		244 (57.8)
**Years in emergency medicine (median [IQR])**	12.00 [7.50, 19.00]	11.00 [8.00, 16.00]	0.304	12.00 [8.00, 17.00]
**Intubation checklist used**	24 (16.8)	81 (29.1)	0.008	105 (24.9)
**Age (median [IQR])**	61 [38, 72]	62 [44.5, 74]	0.587	61 [42, 73]
**Gender = Male (%)**	97 (68.3)	193 (70.2)	0.778	290 (69.5)
**Patient category (%)**				
Trauma Blunt	20 (14.0)	49 (17.9)	0.004	69 (16.5)
Trauma Penetrating	3 (2.1)	5 (1.8)		8 (1.9)
Trauma Head	29 (20.3)	40 (14.6)		69 (16.5)
Trauma Other	3 (2.1)	23 (8.4)		26 (6.2)
Medical Cardiac Arrest	30 (21.0)	81 (29.6)		111 (26.6)
Medical Respiratory	2 (1.4)	12 (4.4)		14 (3.4)
Medical Intoxication	13 (9.1)	9 (3.3)		22 (5.3)
Medical Infection	1 (0.7)	0 (0.0)		1 (0.2)
Medical Other	6 (4.2)	7 (2.6)		13 (3.1)
Neurology Stroke	24 (16.8)	28 (10.2)		52 (12.5)
Neurology Other	11 (7.7)	20 (7.3)		31 (7.4)
Other	1 (0.7)	0 (0.0)		1 (0.2)
**GCS ≤ 8**	111 (79.9)	206 (74.9)	0.318	317 (76.6)
**SBP ≤ 90 mmHg**	15 (12.6)	36 (16.0)	0.494	51 (14.8)
**Riskfactor for difficult intubation (%)**	89 (62.2)	149 (53.4)	0.103	238 (56.4)
**Aggravating conditions (%)**	98 (68.5)	172 (61.6)	0.198	270 (64.0)
**NACA-score (median [IQR])**	6.00 [5.00, 6.00]	5.00 [5.00, 6.00]	0.029	5.00 [5.00, 6.00]
**Prehospital intubation location**				
Outside (e.g. outdoors or indoors at scene)	66 (46.2)	116 (41.6)	0.427	182 (43.1)
In cabin, ambulance or helicopter	77 (53.8)	163 (58.4)		240 (56.9)

**Table 2:** First Pass Success by Airway Assistant category (Anaesthetist (anaesthesiologist or nurse anaesthetist) vs Non-anaesthetist (paramedic, emergency medicine physician, nurse, or other healthcare provider) (IQR, inter quartile range; SD, standard deviation; DL, direct laryngoscope; VL, video laryngoscope; SAD, supraglottic airway device; Cric, cricothyroidotomy; CPR, cardio pulmonary resuscitation)

Airway assistant	Anaesthetist (*n* = 143)	Non-anaesthetist (*n* = 279)	*p*	Overall (*n* = 422)
**First pass success, *n* (%)**	126 (88.1)	245 (87.8)	1.000	371 (87.9)
**Intubation attempts, *n* (%)**				
1 st attempt	126 (88.1)	245 (87.8)	0.279	371 (87.9)
2nd attempt	16 (11.2)	24 (8.6)		40 (9.5)
3rd attempt	1 (0.7)	7 (2.5)		8 (1.9)
4th attempt	0 (0.0)	3 (1.1)		3 (0.7)
**Overall intubation success, *n* (%)**	141 (98.6)	277 (99.3)	0.878	418 (99.1)
**Perceived intubation difficulty, (scale of 1–10) median [IQR]**	2.00 [1.00, 3.00]	2.00 [1.00, 3.00]	0.311	2.00 [1.00, 3.00]
**Intubation time, sec. (SD)**	25.47 (23.84)	22.60 (27.23)	0.292	23.57 (26.15)
**Intubation technique, *n* (%)**				
DL	42 (29.4)	113 (40.5)	0.059	155 (36.7)
VL	99 (69.2)	163 (58.4)		262 (62.1)
SAD	0 (0.0)	2 (0.7)		2 (0.5)
Cric	2 (1.4)	1 (0.4)		3 (0.7)
**Outcome, *n* (%)**				
Alive on arrival	134 (93.7)	263 (94.3)	0.918	397 (94.1)
Ongoing CPR on arrival	5 (3.5)	10 (3.6)		15 (3.6)
Prehospital death	4 (2.8)	6 (2.2)		10 (2.4)
**On-scene time, min. (SD)**	25.47 (23.84)	22.60 (27.23)	0.292	23.57 (26.15)

**Table 3:** Intubation-Related Complications by Airway assistant category (Anaesthetist (anaesthesiologist or nurse anaesthetist) vs Non-anaesthetist (paramedic, emergency medicine physician, nurse, or other healthcare provider) (SpO2, saturation of peripheral oxygen; BP, blood pressure; HR, heart rate; ET, endotracheal tube)

Airway assistant	Anaesthetist	Non-anaesthetist	*p*	Overall
**Post intubation overall complications**	12 (8.4%)	33 (11.8%)	0.32	45 (10.7%)
**Complication category, *n* (%)**				
Hypoxia (SpO2 < 90%)	11 (7.7%)	11 (3.9%)	0.00115	22 (5.2%)
Hypotension (BP < 90 mmHg)	2 (1.4%)	14 (5%)	0.193	16 (3.8%)
Cardiac arrest	0 (0%)	3 (1.1%)	0.562	3 (0.7%)
Bradycardia (HR < 60)	0 (0%)	3 (1.1%)	0.562	3 (0.7%)
ET misplaced in oesophagus (corrected)	0 (0%)	6 (2.2%)	0.32	6 (1.4%)
ET misplaced in oesophagus (not corrected)	0 (0%)	1 (0.4%)	1	1 (0.2%)
ET misplaced in main bronchus	1 (0.7%)	2 (0.7%)	1	3 (0.7%)
Aspiration or vomiting	0 (0%)	1 (0.4%)	1	1 (0.2%)
Surgical airway complications	0 (0%)	0 (0%)	—	0 (0%)
Dental trauma	0 (0%)	0 (0%)	—	0 (0%)

## Results

A total of 422 patients were included in this subgroup analysis. Among these, 143 (33.9%) tracheal intubations were attempted with an anaesthetist (either an anaesthesiologist or nurse anaesthetist) as the airway assistant and 279 (66.1%) were attempted with a non-anaesthetist (paramedic, emergency medicine physician, nurse, or other healthcare provider).

Baseline characteristics by airway assistant category are reported in Table 1. Tracheal intubation attempts with an anaesthetist assistant were significantly more likely to involve more severely ill patients (NACA score 6.0 vs. 5.0 *p* = 0.029) and to not have used checklists (83.2% vs. 70.9%, *p* = 0.008). The non-anaesthetist assistant group had a greater proportion of operators who were an anaesthesiologist (99.3% vs. 91.6%, *p* < 0.001). There were no statistically significant differences observed for patient sex, age, shock or no shock status, or GCS category. In regard to risk for difficult intubation and possibly aggravated conditions, both tended toward more severe conditions in the anaesthetist assistant group than in the control group but did not reach statistical significance. The locations of attempted intubation, in- or outside the cabin of the helicopter or ambulance, were evenly distributed between the groups.

There were no statistically significant differences in FPS between attempted intubations involving anaesthetist assistants and those involving the non-anaesthetist assistants (88.1% vs. 87.8%, *p* = 1.000) (Table 2). The distribution of intubation attempts across categories was similar, with the vast majority of successful intubations achieved on the first or second attempt (99.3% and 96.4%, respectively). The perceived difficulty of intubation, reported on a 10-point scale, was also comparable between the groups (2.00 [1.00–3.00], *p* = 0.311). The mean time to successful intubation did not differ significantly (25.5 vs. 22.6 s, *p* = 0.292). While video laryngoscopy (VL) was the most common technique overall (62.1%), it was more common in the anaesthetist-assisted group than in the non-anaesthetists’ group (69.2% vs. 58.4%), although this difference did not reach statistical significance. The use of rescue techniques (supraglottic airway devices or cricothyrotomy) was rare and did not differ significantly between groups.

Since there was a significant difference in baseline characteristics between the anaesthetist and non-anaesthetist assistant groups where the latter had a greater proportion of operators who were anaesthesiologists (99.3% vs. 91.6%, *p* < 0.001), a separate analysis was performed excluding all other operators to determine whether there was a difference in the primary outcome. The FPS for attempted intubations by anaesthesiologists assisted by anaesthetist and non-anaesthetist were the same, 115/131 (87.8%) vs. 244/277 (88.1%), *p* = 1.00, compared with 126/143 (88.1%) vs. 245/279 (87.8%), *p* = 1.00.

Post-intubation complications are presented in Table 3. The overall complication rate was 10.7% (45/422), with no statistically significant difference between intubations involving anaesthetist assistants and non-anaesthetists (8.4% vs. 11.8%, *p* = 0.32). The most frequently observed complication was hypoxia (SpO₂ < 90%), which occurred in 5.2% of the patients overall. Hypoxia was more common in the anaesthetist assistant group (7.7% vs. 3.9%, *p* = 0.00115). Other complications such as hypotension (3.8%), oesophageal intubation (1.7%), and bradycardia (0.7%) were infrequent and similarly distributed between the groups. No cases of dental trauma or surgical airway complications were reported. Misplacement of the endotracheal tube in the oesophagus occurred in seven patients (1.7%), all of whom were recognized and corrected in six patients.

Multivariate logistic regression was used to assess the associations between airway assistant profession (exposure) and two outcomes: FPS and post-intubation complications. Covariates included in the models were selected a priori.

For the primary outcome of FPS, the adjusted odds ratio (aOR) for intubations involving an anaesthetist assistant, compared with non-anaesthetists, was 1.05 (95% CI 0.54–2.12), indicating no statistically significant association (Fig. 1). Among all included variables, the only factor significantly associated with higher likelihood of FPS was the presence of an assessed no difficult airway predictor (aOR 3.43, 95% CI 1.71–7.51). The use of video laryngoscopy and greater operator experience were associated with numerically greater odds of FPS, although these associations did not reach statistical significance. Variance inflation factors (VIF) were inspected for multicollinearity, all values were < 2.0, indicating no serious collinearity issue.

For the secondary outcome of post-intubation complications, there was no independent association between assistant profession and overall complication risk (aOR for non-anaesthetist: 1.79, 95% CI 0.66–5.39; Fig. 2). However, increased complication risk was observed in patients with systolic blood pressure ≤ 90 mmHg (aOR 3.52, 95% CI 1.56–8.19) and in patients who were intubated outside the cabin (aOR 2.47, 95% CI 1.14–5.42). Checklist non-use and direct laryngoscopy were also associated with elevated complication rates, but with wider confidence intervals and uncertain precision.

While the crude OR for FPS with an anaesthetist assistant, compared with a non-anaesthetist, was 1.03, the adjusted OR increased to 1.05 (95% CI 0.54–2.12), suggesting a non-significant trend but no evidence of a clear benefit once confounders were accounted for. For complications, the crude OR for non-anaesthetist assistants was 1.46, and the adjusted OR rose to 1.79 (95% CI 0.66–5.39), again indicating a non-significant association with a wide confidence interval. These shifts between crude and adjusted estimates suggest possible confounding, but the width and overlap with 1.0 reflect limited precision and statistical uncertainty.

## Discussion

In this subgroup analysis of the PHASTER study data, we found no statistically significant associations between the airway assistant profession and FPS or complication rates during prehospital drug-assisted tracheal intubations. These findings persisted even after adjusting for multiple covariates, including airway features, intubation techniques and conditions, and patient characteristics.

While previous studies have highlighted the importance of team composition in airway management [13, 14], we found no studies that have explicitly examined the assistant’s role. Our study addresses this specific knowledge gap and shows that among highly trained Scandinavian HEMS teams, the assistant’s professional background, whether anaesthetist or non-anaesthetist, does not appear to influence FPS (aOR 1.05, 95% CI 0.54–2.12) or complication risk (aOR 1.79, 95% CI 0.66–5.39).

This lack of observed difference warrants interpretation. First, both groups of assistants had intubation operators with high baseline competence: the majority of operators had substantial intubation experience (> 1000 prior intubations in 88.6% of cases) and a median of 12 years in pre-hospital emergency medicine, working in mature systems with strong procedural governance. This aligns with data from Gellerfors et al. (2018), who reported similarly high FPS in Scandinavian HEMS regardless of staff background [15].

Second, mutual familiarity and shared mental models may buffer the effect of professional background. Garcia et al. (2024) used video reflexive ethnography during real-time emergent intubations and reported that relational dynamics, “trust, anticipation, and communication”, strongly shaped procedural success, often more than individual role expertise​ [16]. In our cohorts, team structure may have been more homogeneous in practice than the labels 'anaesthetist' and 'non-anaesthetist' suggest.

Third, it is possible that team-wide standardization through structured protocols, pre-allocated roles, and checklists diluted the variability between assistant types. However, notably, checklist use was significantly lower in the anaesthetist group (16.8% vs 29.1%, *p* = 0,008), perhaps reflecting greater task familiarity. However, the outcomes remained similar, suggesting compensatory mechanisms such as implicit coordination or greater informal task division.

Our findings also align with the previously described Inter-Changeable Operator Model (ICOM). In this model, developed within UK HEMS, operator and assistant roles can be interchanged without compromising FPS when the primary operator is highly skilled [17]. Similar results have been reported in an Australian HEMS, where the ICOM approach yielded comparable success rates and complication profiles across role allocations [18]. These data support the interpretation that the value of an assistant may diminish when the intubation operator is an experienced anaesthesiologist. However, when the primary operator is a non-physician, evidence consistently shows lower success rates [19]. In such settings, an experienced anaesthesiologist assistant may play a critical compensatory role, highlighting that the impact of assistant background is context-dependent and influenced by both operator expertise and system design.

Over the past few decades, the standardization of airway management and reporting has significantly improved. The revised Utstein-style template for reporting prehospital airway interventions provides a structured framework for capturing key variables such as time, patient factors, airway management and system descriptors [6]. This has supported a more consistent reporting of outcomes and has been further refined with the developed quality indicators (QI) for prehospital advanced airway management [20]. One such commonly used QI is first-pass success (FPS), which is successful tracheal intubation on the first attempt. While FPS has been associated with reduced complication rates and faster procedural times [1, 15], it is increasingly recognized as a surrogate outcome, rather than a measure of patient benefit [21].

Moreover, there has been a growing shift in the literature from evaluating individual technical skills to understanding team performance as the primary driver of success in potentially demanding procedures such as emergency intubations. Simulation-based studies and quality improvement initiatives have emphasized the importance of using checklists, good CRM, and training teams. Gopinath et al., for example, demonstrated that improving team dynamics and workflow in an emergency department setting significantly reduced intubation time, even when no changes were made to personnel or equipment [22]. Similarly, a systematic review by Garner et al. on paediatric prehospital intubation revealed that physician-led teams had the highest FPS and lowest complication rates, suggesting that success is mediated by both skill level and team composition [13].

To further support quality improvement in prehospital airway care, structured and reproducible approaches have been proposed that emphasize team readiness, predefined roles, and escalation strategies. One example is the Vortex approach, which aims to improve consistency and safety in airway decision-making by promoting shared mental models, closed-loop communication, and timely transitions between techniques [23]. Such frameworks shift the focus from isolated technical performance to team success in airway management, reinforcing the concept that procedural outcomes emerge from coordinated team dynamics rather than individual action alone.

When comparing our findings to those from paediatric and mixed-setting studies, a more nuanced picture emerges. Garner et al. (2020) reported that in prehospital paediatric intubation, team composition, specifically physician-led teams, was associated with higher FPS and fewer complications​ [13]. However, that study involved more varied operator backgrounds, and the assistants’ role was not reported. In contrast, our study offers more targeted insight by specifically stratifying results by assistant profession while holding operator background (mostly anaesthesiologists) constant.

It is also worth considering why no benefit of anaesthetist-assistants was observed, despite assumptions about their superior airway knowledge. One hypothesis is that during high-stakes intubation, the most critical decisions (e.g., initiated resuscitation, induction dosing, airway technique selection) remain physician-driven. If assistants primarily support logistical or manual tasks (e.g., drug preparation, airway adjunct assistance), then their influence may be secondary in high-functioning teams. Indeed, studies by Howarth (2016) and Hersey et al. (2017) both suggest that assistants impact scales with empowerment and specific task ownership, which may not vary as much by profession in real-world practice [24, 25].

Interestingly, hypoxia occurred more frequently in the anaesthetist-assisted group (7.7% vs. 3.9%, *p* = 0.00115), despite otherwise balanced patient characteristics. While speculative, this may be related to subtle case-mix differences, e.g., more severely ill patients in the anaesthetist group (NACA score 6.0 vs. 5.0, p = 0.029). It may also reflect differential vigilance in recognizing or reporting complications, a known source of variation in prehospital airway datasets [20].

Our findings align with a growing body of literature emphasizing that team structure, CRM, and role experience are as important as operators’ technical ability in airway management. As stated above, structured team workflows significantly improved intubation performance even without changing team personnel, and in paediatric prehospital care it has been shown that FPS was highest in teams with consistent training models, particularly physician-led teams [13]. This echoes findings from HEMS systems such as the Greater Sydney and London HEMS, where cross-role competence and SOP compliance are prioritized over rigid hierarchies [3, 4].

While FPS is a widely accepted process metric, its limitations must be acknowledged. This is correlated with fewer complications, shorter procedural times, and lower operator stress [1, 5]. However, a large Finnish HEMS registry study revealed that the FPS did not predict 30-day survival, supporting its role as a mere surrogate marker rather than a patient-centred outcome [21]. Our results suggest that FPS, in isolation, does not fully capture the quality of prehospital airway management and should be considered within a multidimensional performance framework and therefore should be included in composite outcome measures together with patient mortality and/or morbidity. Kottmann et al. recently outlined consensus-based quality indicators for prehospital airway management, emphasizing structured preparation, timely execution, and system-level readiness [20]. Our findings in a Scandinavian context support this direction: when systems maintain procedural standards, uphold continuous quality improvement programs, and include anaesthesiologists, performance outcomes may be robust even when the assistant role varies.

## Limitations

This study has several limitations. First, it may be underpowered to detect subtle differences in FPS or complication rates between airway assistant groups. With only 45 cases with complications and relatively balanced FPS rates, the statistical power to detect small effect sizes is limited. Second, we analysed only included and registered intubation attempts, which may introduce selection bias, perhaps excluding challenging or aborted attempts that never were registered. Third, despite multivariable adjustment, the observational design of the study implies a risk of residual confounding, particularly regarding unmeasured variables such as years of experience with assistants, team familiarity, and inter-provider communication quality. In the logistical regression the absence of statistically significant differences, particularly given the low number of complications, should not be interpreted as evidence of equivalence between groups. Although the number of complication events was limited, the FPS model included approximately 50 events, which corresponds to 10 events per covariate. This is consistent with commonly cited thresholds for logistic regression stability. The selection of covariates prioritized clinical relevance rather than purely statistical optimization, in line with recommendations for observational airway research. Wide confidence intervals in some subgroups likely reflect the low number of events rather than model instability. Penalized regression approaches were considered but deemed unnecessary, as results remained stable when variables were added or removed. Moreover, as these regression models were designed to examine associations, not predictions, no conclusions can be drawn regarding their discriminative ability.

Finally, our findings are derived from anaesthesiologist-staffed Scandinavian HEMS systems with highly experienced clinicians and high baseline performance which is why generalizing these results to other EMS systems with differing team compositions or airway expertise might not be applicable.

Nonetheless, the study benefits from prospective data collection, multicentre inclusion, and adherence to the updated Utstein-style template, making it possibly the most comprehensive investigation to date on the impact of airway assistants on prehospital intubation success.

## Conclusion

In this multicentre observational analysis of prehospital advanced airway management, the profession of the airway assistant was not independently associated with FPS or complication rates. Taken together, our findings suggest that in the specific setting of well-trained HEMS teams operating under standardized protocols, the professional background of the airway assistant may be less important. Future research should continue to explore how team design and simulation-training can optimize prehospital airway management across various HEMS settings.

## Data Availability

No datasets were generated or analysed during the current study.
